# Processing of oat: the impact on oat's cholesterol lowering effect

**DOI:** 10.1039/c7fo02006f

**Published:** 2018-02-12

**Authors:** Myriam M.-L. Grundy, Anthony Fardet, Susan M. Tosh, Gillian T. Rich, Peter J. Wilde

**Affiliations:** a Food and Health Programme , Quadram Institute Bioscience , Norwich Research Park , NR4 7UA , UK . Email: myriam.grundy@quadram.ac.uk ; Email: wildongillian@gmail.com ; Email: peter.wilde@quadram.ac.uk ; Tel: +44 (0)1603 255258; b INRA , JRU 1019 , UNH , CRNH Auvergne , F-63000 Clermont-Ferrand & Université de Clermont , Université d'Auvergne , Unité de Nutrition Humaine , BP 10448 , F-63000 Clermont-Ferrand , France . Email: anthony.fardet@inra.fr; c University of Ottawa , Université , Salle 118 , Ottawa , ON K1N 6N5 Canada . Email: Susan.Tosh@uottawa.ca

## Abstract

Epidemiological and interventional studies have clearly demonstrated the beneficial impact of consuming oat and oat-based products on serum cholesterol and other markers of cardiovascular disease. The cholesterol-lowering effect of oat is thought to be associated with the β-glucan it contains. However, not all food products containing β-glucan seem to lead to the same health outcome. Overall, highly processed β-glucan sources (where the oat tissue is highly disrupted) appear to be less effective at reducing serum cholesterol, but the reasons are not well understood. Therefore, the mechanisms involved still need further clarification. The purpose of this paper is to review current evidence of the cholesterol-lowering effect of oat in the context of the structure and complexity of the oat matrix. The possibility of a synergistic action and interaction between the oat constituents promoting hypocholesterolaemia is also discussed. A review of the literature suggested that for a similar dose of β-glucan, (1) liquid oat-based foods seem to give more consistent, but moderate reductions in cholesterol than semi-solid or solid foods where the results are more variable; (2) the quantity of β-glucan and the molecular weight at expected consumption levels (∼3 g day^–1^) play a role in cholesterol reduction; and (3) unrefined β-glucan-rich oat-based foods (where some of the plant tissue remains intact) often appear more efficient at lowering cholesterol than purified β-glucan added as an ingredient.

## Abstract 2

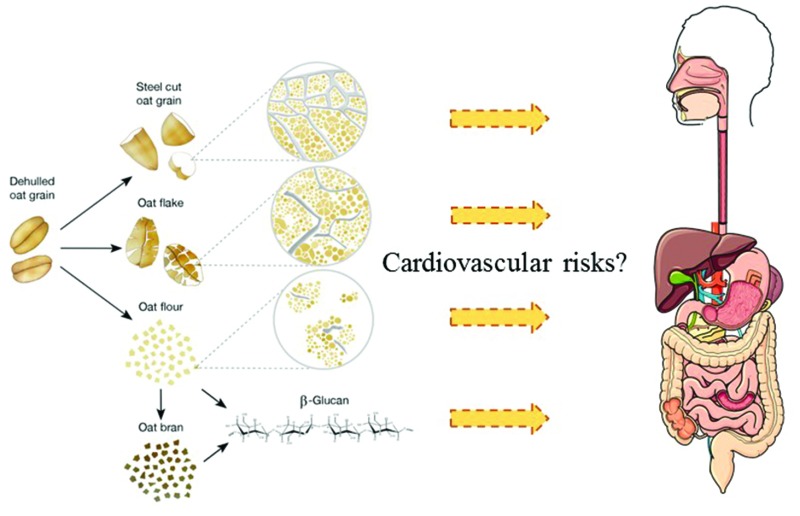
Human studies have clearly demonstrated the beneficial impact of consuming oats on cholesterol levels, however, processing can have a significant influence on functionality, which has not yet been fully addressed.

## 1. Introduction

It is widely recognized that the intake of dietary fibre in a typical Western diet is below recommendations. Oat is one of several grains eaten as part of a Western diet but its consumption and global production are much lower compared to the staple crops wheat, maize, rice, and barley.^1^ One of the reasons for its low global production could be a lack of diversity in the oat products commercially available. However, studies reveal the multiple beneficial effects on health associated with oat consumption, ranging from reduction in risk of cardiovascular diseases^2^–^4^ to cancer prevention.^5^,^6^ Compared with other cereals, oats have higher concentrations of certain nutrients and phytochemicals (*e.g.*, essential amino acids and fatty acids, β-glucan and phenolic compounds), and they can tolerate harsher growing conditions such as wet climate and acidic soil, making them more resilient than other crops.^7^

In the ageing population of Western countries, cardiovascular disease (CVD) and related conditions are a huge public health burden. According to the World Health Organisation (WHO), CVD is the number one cause of death globally. For example, in 2015, 17.7 million people died from CVD. Elevated serum cholesterol (hypercholesterolaemia) is a significant risk factor for developing CVD. Hypercholesterolaemia can be treated by prescribing statins but this therapy is associated with various negative side-effects. Diet is a key risk factor for the development and prevention of CVD.^8^ Thus, using dietary approaches that tackle risk factors such as increased serum cholesterol levels and high systolic blood pressure should be a key strategy for the prevention of CVD and other metabolic disorders.

The first study to reveal that oat consumption reduced plasma cholesterol levels goes back to 1963.^9^ By enriching bread with rolled oat, the authors observed an 11% reduction in total cholesterol levels. Since then a multitude of *in vivo* and *in vitro* investigations have been conducted to understand the reasons behind this positive effect.^2^–^4^,^10^–^15^ To date, the cholesterol-lowering effect of oat has been attributed primarily to the β-glucan it contains.^2^,^3^,^10^,^13^,^14^ Although the precise mechanisms involved are not completely understood, the ability of β-glucan to lower cholesterol is thought to be triggered by several processes.^11^,^16^ Firstly, the presence of β-glucan in the small intestine may increase the viscosity of intestinal contents, which could delay gastric emptying, reduce intestinal mixing, entrap mixed micelles, and restrict the mixing and transport of nutrients, digestive enzymes and bile salts.^17^ Secondly, it has been hypothesised that β-glucan could also interfere with the enterohepatic recycling of bile salts by direct interaction.^11^ However, the effect of β-glucan on bile salt metabolism still remains unclear as recently demonstrated by the same authors.^18^,^19^ Finally, β-glucan has been shown to interact with the mucus layer resulting in a diminution in the porosity of the intestinal mucus and thereby a reduction in nutrient absorption.^20^

Following a review of some of the human studies on the subject, health claims were approved in the United States in 1997 by the Food and Drug Administration (FDA),^21^ and later in Europe by the European Food Safety Authority (EFSA)^22^ and in Canada. These claims stipulated that foods should provide at least 3 g per day of β-glucans in order to obtain the claimed effect. The EFSA regulation stipulates that oats should be consumed in a minimally processed form. Indeed, not all food products containing β-glucan seem to lead to the same health outcome; overall processes that degrade β-glucan result in products that are less effective at reducing plasma cholesterol.^23^

Current knowledge suggests that the complexity and structure of the matrix or interactions between different components are key factors controlling functionality.^24^–^27^ Thus far, few studies have fully characterised the physicochemical properties of oat or oat derived ingredients (*e.g.*, detailed chemical analysis of nutrients, integrity of cell walls, rheology, location and physical state of starch granules, protein bodies and oil bodies, and β-glucan molecular weight and content), making interpretation of these findings difficult. In addition, conflicting findings might be notably the consequence of a reductionist, nutrient-focused (*i.e.*, β-glucan) approach.^28^,^29^

This review will focus on the influence of the structure and complexity of the matrix of oat and oat-based foods on their nutritional impact in order to provide information for future research on the mechanisms influencing the beneficial health attributes of oat.

## 2. The oat matrix and its impact on cholesterol and risk of cardiovascular disease

### 2.1 Oat structure and composition

**Fig. 1:**
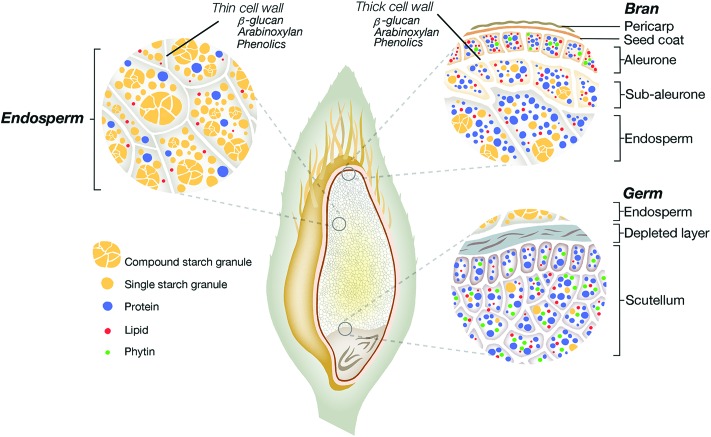
Structural representation of the oat grain presenting different oat tissues (*i.e.*, the bran, germ and endosperm) and the nutrient distribution/organisation within these tissues.

A food matrix can be described as the structure and hierarchical organisation (from molecule to tissue) of the components of a food product.^26^ The spatial arrangement and the relationship between the components constitutive of the plant food matrix can occur naturally (*e.g.*, cellular structure of plant tissues) or result from processing (*e.g.*, network structure developed during breadmaking). The oat grain is a complex matrix containing the protective hull and the groat (caryopsis), the latter being composed of the bran, germ and starchy endosperm (Fig. 1).^30^ The bran is a coarse outer layer rich in minerals, vitamins, and cell wall polysaccharides, mainly cellulose, arabinoxylan and β-glucan. Below the pericarp and seed coat of the bran, there are the aleurone and subaleurone layers joined to the endosperm. The aleurone and subaleurone cells are surrounded by thick cell walls resistant to digestion, whereas the endosperm cells have thinner cell walls, rich in β-glucan. The oat protein and lipid increase in concentration from the interior to the periphery of the groat, while starch increases from the subaleurone region to the centre of the endosperm.^30^ The structure and composition of the proteins (globulins, albumins, prolamins (avenin) and glutelins) differ depending on the part of the grain in which they are located.^31^ Two types of starch granules exist in the endosperm: compound and single granules.^30^ The proportions of amylose and amylopectin, and the size of the granules vary between oat varieties.

Oat is potentially a good source of macronutrients, vitamins, minerals, and other phytochemicals, but the actual dietary intake depends on the part of the grain (*i.e.*, endosperm *vs.* bran) that is consumed and how it has been processed.^32^,^33^ The proportion of the different nutrients can also be altered.^34^ One common practice employed by manufacturers to increase the β-glucan content of oat products is to add high β-glucan content grain fractions.

**Fig. 2:**
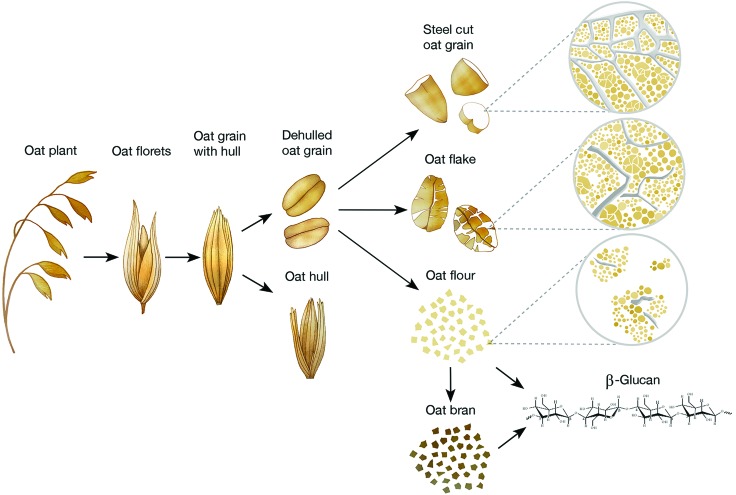
Diagram showing the structural levels of oat, from the plant to extracted β-glucan, and some oat forms commonly consumed (*i.e.*, steel cut oat grain, oat flakes, oat flour, oat bran and purified β-glucan).

Fig. 2 summarises the different levels of oat structural complexity, from the plant to the purified β-glucan, including oat products commonly consumed. The oat ingredients frequently found in the literature are bran, purified β-glucan, flakes (also called oatmeal or rolled oats) and flour. Rolled oats exist in a wide range of particle sizes (from ∼0.2 to a few mm; *e.g.*, large flake, Irish steel cut, Scottish, quick and instant oat flakes).^35^,^36^ Oat bran is produced by grinding and sieving to remove part of the starch and is available in a range of particle sizes. According to the definition from the American Association for Cereal Chemistry, oat bran must contain a minimum of 5.5% β-glucan^34^,^37^ but fractions are available with β-glucan contents up to 40%.^35^,^36^ They can then be incorporated into other food products, such as breakfast cereals (*i.e.*, ready-to-eat cereals, muesli, and granola), biscuits and cookies, cereal bars, bread, and muffins.^36^

Oat extracts are made by extracting the β-glucan using enzymatic and/or solubilisation methods. Several extraction methods have been patented including those that use amylases,^38^–^40^ freeze/thaw fractionation,^41^ and solubilisation with basic solutions.^42^ These purification methods can achieve much higher β-glucan concentrations than dry milling methods. For more details of the protocols and advantages/disadvantages of each method, the readers are referred to earlier articles.^12^,^43^

### 2.2 Oats and their cholesterol-lowering effect

In this review, we chose to investigate the effect of oat and oat-based products on lipemia (cholesterol and triglycerides) and other cardiovascular risk factors based on the differences in food matrices in order to correlate differential effects according to the physicochemical characteristics of these matrices. It has been established that subjects with mildly elevated cholesterol levels have to consume at least 3 g of β-glucan per day to observe a significant reduction in their serum total- and low-density lipoprotein- (LDL) cholesterol levels.^3^ A study that compared the efficacy of extruded oat bran cereals, which underwent graded increases in the temperature and pressure used in the extrusion process to modify the β-glucan so that they had a range of molecular weights, indicated that the increased viscosity in the gastrointestinal tract played a role in the mechanism of action.^44^ The increase in viscosity prevents efficient mixing of the luminal contents, which results in decreased intestinal uptake of dietary cholesterol and decreased reabsorption of bile acids.^45^ β-Glucan can interfere with the enterohepatic circulation at different levels including limiting the return of bile acids to the liver, which triggers an increase in hepatic conversion of cholesterol into bile acids and thereby a decrease in blood cholesterol levels.^46^

Numerous oat products have been used as a source of β-glucan, in both *in vitro* and *in vivo* studies. The most studied products or ingredients are purified β-glucan, oat bran, oat flakes and oat-based food products. The latter were made of oat that underwent various degree of processing, and may have contained part of the oat tissue (*e.g.*, bran, flour and flakes) or only certain oat constituents (*e.g.*, β-glucan). Table 1 presents a summary of the human studies mentioned in sections 2.2.1 to 2.2.4 based on product type.

**Table 1:** A summary of some of the human studies on the effect of oat-based products on lipemia

Ref.	Process	Oat-based products	Dose (per day)	Texture	TC (%)	LDL-C (%)	TAG (%)
Bremer (1991)^85^	Fermentation Baking	Oat bran bread	44.6 g of oat bran	Solid	NS	NS	NS
Frank (2004)^86^		Yeast-leavened oat bran breads containing BG of low or high average molecular weight	6 g of BG	Solid	NS	NS	nd
Kerckhoffs (2003)^87^		Oat bran bread	5.9 g of BG	Solid	NS	NS	nd
Liatis (2009)^81^		Oat flour bread	3 g of BG	Solid	–13	–16	–12
Reyna-Villasmil (2007)^79^		Oat bran bread	6 g of BG	Solid	–16	–28	NS
Zhang (1992)^80^		Oat bran bread	118 g of oat bran, 29 g of DF	Solid	–11	–15	nd

Anderson (1990)^88^	Breakfast cereals	Oat bran cereals (flakes, biscuits or ready-to-eat cereals)	25 g of oat bran, 8.8 g of DF	Solid	–5	–9	nd
Bartram (1992)^77^		Oat bran cereal muesli	60 g of oat bran	Solid	–11	–11	nd
Beck (2010)^68^		Oat cereals (ready-to-eat cereals or flakes, and muesli bars)	5–6 or 8–9 g of BG	Solid and semi-solid	NS	NS	NS
Bindu (2013)^89^		Oat flakes	35 g of oat	Semi-solid	NS	–8	NS
Charlton (2012)^63^		Oat cereals (ready-to-eat cereals, flakes and snack bars)	1.5 or 3 g of BG	Solid	NS	–18	NS
Davy (2002)^64^		Oat flakes and ready-to-eat cereals	5.5 g of BG	Solid and semi-solid	NS	NS	NS
Gold (1991)^78^		Ready-to-eat oat bran cereals and snack bars	38 g of oat bran	Solid	NS	NS	nd
Lovegrove (2000)^90^		Oat bran incorporated into ready-to-eat cereals	3 g of BG	Solid	NS	NS	NS
Maki (2010)^91^		Whole-grain ready-to-eat oat cereals	3 g of BG	Solid	–9	–5	NS
Marlett (1994)^92^		Oat bran cereals	6.9 g of BG	Solid	—	nd	nd
Pins (2002)^65^		Oat flakes and ready-to-eat cereals	5.7 g of BG	Solid	–15	–16	nd
Thongoun (2013)^69^		Oat flakes	70 g of oat	Semi-solid	–5	–10	NS
Van Horn (1988)^66^		Oat flakes	56 g of oat	Semi-solid	–8	—	—
Van Horn (1991)^93^		Oat flakes	56.7 g of oat, 5.6 g DF	Semi-solid	–6	–9	—
Van Horn (2001)^67^		Oat flakes	56 g of oat	Semi-solid	–5	–5	NS
Wolever (2010)^23^		Oat bran incorporated into ready-to-eat cereals	3 or 4 g of BG	Solid	NS	–6	NS
Zhang (2012)^70^		Oat flakes	100 g of oat	Semi-solid	–7	–9	NS

Amundsen (2003)^94^	Other products	Premade diet containing oat bran concentrate in 8 products (muesli, extruded breakfast flakes, bread, teacakes, muffins, tagliatelle pasta, macaroni and an apple drink)	5 g of BG	Solid and liquid	–6	–9	nd
Chen (2006)^95^		Oat bran concentrate incorporated into muffins and ready-to-eat cereals	7.3 g of BG	Solid	NS	NS	NS
Romero (1998)^84^		Cookies enriched with oat bran	2.6 g of BG	Solid	nd	–26	NS
Stewart (1992)^96^		Oat bran	51.7 g of oat bran	Solid	NS	NS	NS
Swain (1990)^97^		Muffins and entrées enriched with oat bran	100 g of oat bran	Solid	NS	NS	NS
Uusitupa (1997)^61^		Oat bran	10.3 g of BG	Solid	NS	—	—
Whyte (1992)^62^		Oat bran	123 g of oat bran	Solid	–3	–6	—

Beer (1995)^98^	Beverages Soups	Oat gum instant whip	9 g of BG	Semi-solid	NS	NS	NS
Biorklund (2005)^56^		BG (70 kDa) incorporated into beverages	5 or 10 g of BG	Liquid	–7	–6	NS
Biörklund (2008)^99^		BG (80 kDa) incorporated into soup	4 g of BG	Liquid	NS	NS	NS
Cugnet-Anceau (2010)^100^		BG (80 kDa) incorporated into soup	3.5 g of BG	Liquid	NS	NS	NS
Ibrugger (2013)^101^		BG (305 kDa) incorporated into beverages	3.3 g of BG	Liquid	NS	NS	NS
Kerckhoffs (2003)^87^		Oat bran in orange juice	5.9 g of BG	Liquid	–4	–7	nd
Mårtensson (2005)^102^		Fermented oat-based products	3 or 3.6 g of BG	Liquid	–6	–6	nd
Naumann (2006)^103^		BG incorporated into a fruit drink	5 g of BG	Liquid	–3	–5	NS
Onning (1998)^104^		Oat milk		Liquid	–4	–9	nd
Onning (1999)^105^		Oat milk	3.75 g of BG	Liquid	–6	–6	NS

#### 2.2.1. Purified β-glucan

A few studies have investigated the cholesterol lowering potency of purified forms of β-glucan. Several methods exist to extract the polymer from the oat tissue with consequences on its molecular weight and content.^12^ It is actually challenging to obtain pure β-glucan extracts of both high concentration and molecular weight. Purified oat extracts were originally called oat gums.^47^ Gums can be described as water-soluble fractions of plant, fungal, animal or synthetic origin, rich in a particular polysaccharide.

Consumption of 2.9 g per day of oat extract by mildly hypercholesterolemic humans resulted in a 9% decrease in LDL cholesterol levels compared to the placebo (maltodextrin) phase of the trial.^48^ Interestingly, when oat extract was discontinued, total and LDL cholesterol returned to initial levels in one study with hypercholesterolemic male and female adults. In rats fed an oat extract, cholesterol uptake by everted jejunal sacs was progressively inhibited by increasing the concentrations of oat gum in the mucosal medium.^49^ Other studies have shown that oat extracts can reduce cholesterol and LDL cholesterol oxidation *in vitro*. This oxidation is thought to be involved in the first steps of atherosclerosis.^50^,^51^ Cholesterol oxidation *in vitro* was also inhibited in a dose-dependent manner upon the addition of oat extracts.^52^ These antioxidant effects are thought to be due to the phenolic compounds present in the oat extracts.

In rats, the effect of native β-glucan as well as β-glucan hydrolysates (product of acidic or enzymatic β-glucan hydrolysis resulting in polysaccharides of smaller molecular weight) on cholesterol and lipid metabolism was investigated.^53^,^54^ Both supplements significantly reduced the levels of LDL and very low-density lipoprotein (VLDL) cholesterol in serum and further improved the lipid profile in liver.^53^ Greater serum triglyceride reduction was observed with β-glucan hydrolysate of an average molecular weight of 730 kDa (*vs.* native) but total cholesterol reduction was insignificant with both β-glucans. In addition, the hydrolysate was more effective at increasing the excretion of faecal cholesterol and triglyceride than the native β-glucan, showing its effectiveness in improving the lipid profile. In contrast, in mice fed a high-fat diet supplemented with β-glucan of three different molecular weights obtained through enzymatic hydrolysis (1450, 730, and 370 kDa), significant reductions in LDL cholesterol resulted, with no effect of molecular weight on the serum lipid profile.^54^ The main limitation of these studies was the extremely large amount of β-glucan given to the rodents (∼8.5 g of β-glucan per day), which is far in excess of any published human study (accounting for body weight and meal size). The large doses administered would result in high viscosity, even for low molecular weight samples, which could also have interfered with the digestibility of the matrix and thereby serum lipid and cholesterol levels. Therefore, caution must be taken in extrapolating these results to humans.

In hypercholesterolemic rats fed diets containing oxidized β-glucan, the levels of triglyceride, total cholesterol, LDL cholesterol, and VLDL cholesterol significantly decreased and more faecal elimination of total cholesterol and triglyceride was observed – in correlation with their reduced levels in serum and liver.^55^ The authors also showed that oxidation of the β-glucan increased its bile acid sequestration (“binding”) capability. Although this effect was relatively small, this interaction between the oat matrix and the bile acids could contribute to the observed increase in fat and cholesterol excretion. In addition, the oxidation significantly increased the water solubility of β-glucan by modifying the polarity of the polymer. This would result in a higher water holding capacity and increased viscosity, which could also support the hypothesis linking luminal viscosity with reduced lipid digestion and uptake, leading to reduced serum cholesterol levels as described earlier in section 2.2.

Beverages enriched with 5 g of oat β-glucan (molecular weight of 70 kDa)^56^ decreased the levels of total plasma cholesterol in hyperlipidemic subjects. However, the reductions in LDL cholesterol were not significant compared to controls (where the LDL cholesterol unaccountably increased during the study, which is a major issue) but were significantly lower than baseline measurements. Interestingly, larger amounts of β-glucan did not necessarily lead to a higher impact on lipemia since the beverage with 10 g of β-glucan from oat did not affect serum lipids significantly in comparison with a control with no added β-glucan.

Overall, purified β-glucan administered under different forms can lead to a small decrease in cholesterol levels, but the results appear inconsistent between studies, which may be due to the treatment applied to the β-glucan.

#### 2.2.2. Oat bran

Oat bran as such has been tested in animal models and in humans. In the most recent study performed on mice, all oat bran preparations significantly reduced plasma cholesterol when compared with a cellulose-containing control diet, regardless of the molecular weight of β-glucan (2348, 1311, 241, 56, 21 or <10 kDa).^57^ In this study, the difference in viscosity between the processed oat brans did not appear to play a major role in the cholesterol-lowering properties. However, the dose given to the mice was 6.8–8.4% β-glucan so that even the diets containing low molecular weight β-glucan were capable, according to the authors, of generating a significant increase in viscosity in the gut.

In two studies performed in hamsters, total plasma cholesterol was significantly reduced (14% and 27%) by oat bran (∼3 g of β-glucan) as compared with the cellulose control diet.^58^,^59^ Then, Jackson *et al.* showed in rats that oat bran significantly reduced the plasma cholesterol level and that in all animals fed oat bran, the liver cholesterol level was lower than in rats fed barley or malted barley.^60^ Studies in humans using oat bran (Table 1) also reported plasma cholesterol reduction but to a lesser extent (6%) than in animals. However, as indicated above, the discrepancy might be due to the high dose of oat bran administered to the rodents (about 3 g of β-glucan for an animal weighing on average between 45 and 60 g, compared with 3 to 10 g of β-glucan for a human presumably weighing on average between 50 and 80 kg).^61^,^62^

#### 2.2.3. Oat flakes

Different diets (*e.g.*, hypocalorific or rich in various sources of oat β-glucan) that include oat flakes were found to improve the plasma lipid levels of hypercholesterolemic subjects.^63^–^68^ Charlton *et al.* and Pins *et al.* reported the largest decrease in LDL cholesterol: 18 and 16%, respectively.^63^,^65^ However, these studies also included other, processed foods such as snack bars and extruded cereals, so the influence of these elements cannot be excluded. Other clinical trials looking specifically at the impact of oat flakes on cholesterol also found a reduction in both total (about 6%) and LDL (about 9%) cholesterol.^69^,^70^ In these investigations, the oats were administered in a semi-solid form where flakes were cooked with some liquid (milk or water).

#### 2.2.4. Oat-containing foods

Oat-based products or β-glucan enriched products have been generally tested in humans or animals for their ability to reduce plasma cholesterol, in particular, LDL cholesterol levels.^2^,^3^,^13^ The other cardiovascular risk factors studied *in vivo* were anti-atherogenic potential,^71^ inhibition of cholesterol and fatty acid oxidation,^51^ blood pressure,^72^–^74^ endothelial function,^75^ fibrinogen,^76^ and apolipoprotein.^77^,^78^

Oat may be incorporated into various matrices such as fermented oat-based products (including breads), oat-based breakfast cereals, pastries, beverages, oat-bran-enriched products and also in the form of oat extracts such as oat gum or concentrates. We reviewed cholesterol and LDL cholesterol reductions in humans according to these different matrices. There is no marked trend relating to matrices, whether they are solid (*e.g.*, oat bran bread), liquid (*e.g.*, oat milk) or semi-solid (*e.g.*, oat porridge and oat gum instant whip) (Table 1). Therefore we cannot really confirm the conclusion of Othman *et al.* that reduction in cholesterol levels may be greater when β-glucan has been added to a liquid compared to a solid food matrix.^13^ This will depend on the solubilisation or dispersion of β-glucan from the food matrix during either preparation, processing or consumption and digestion (see sections 3.1 and 3.2).

As for solid, fermented oat-based products (*e.g.*, bread), their consumption in humans led to cholesterol reduction up to 16% and LDL cholesterol reduction up to 28% but no effect on plasma triglycerides.^79^–^81^ The influence of β-glucan hydrolysis has been studied in ileostomy subjects consuming oat bran bread with and without added β-glucanase.^82^ The bread containing oat bran led to a 53% higher bile acid excretion than oat-bran bread with β-glucanase, probably explaining the effect of oat fibre in lowering the levels of serum lipids. However, the results can be inconsistent between studies, with some of them showing no significant effect (Table 1).

Concerning oat-based breakfast cereal, involving more intensive technological processes than fermentation (*e.g.*, extrusion), cholesterol reduction may reach 15% and LDL-cholesterol reduction 16%, while no triglyceride reduction was observed (Table 1). In the most recent study, oat cereal products providing either a total of 3 g high-molecular-weight (2250 kDa), 4 g medium-molecular-weight (850 kDa), 3 g medium-molecular-weight (530 kDa) or 4 g low-molecular-weight (210 kDa) oat β-glucan were given for 4 weeks to 367 men and women aged 35 to 70 years with body mass index ≥18.5 and ≤40.0 kg m^–2^.^44^ The authors observed that, while high-molecular weight and medium-molecular weight oat cereal decreased LDL-cholesterol, low molecular weight had no effect. This decreased beneficial effect with low molecular weight β-glucan was confirmed by the same research team in a similar study using an extruded cereal containing β-glucans. Those with molecular weights of 2210 kDa and 530 kDa lowered the LDL cholesterol, globulins, albumins, prolamins (avenin) and glutelins by around 5%. The efficacy was reduced by 50% when the molecular weight was reduced to 210 kDa.^23^ In another study in hamsters, bran from oats was cooked in a twin-screw extruder at either high or low energy input but in this particular case, extrusion did not alter its hypocholesterolemic effects.^83^

Concerning other processed foods there are only a small number of studies showing no convincing or inconsistent effects on cholesterol reduction (Table 1). Apart from one study that revealed 26% reduction of LDL-cholesterol by cookies enriched with oat bran,^84^ in another clinical trial, plasma cholesterolemia was not measured but cholesterol absorption was evaluated isotopically in six ileostomy subjects following test meals composed of thick pancakes made primarily from commercially available oat bran or wheat flour and served with crème fraiche and apple sauce.^45^ The results showed that the postprandial cholesterol concentration in the chylomicrons was reduced by 43% after the oat bran test meal was consumed, indicating a decrease in the amount of cholesterol that gets transported from the intestine to other tissues in the body, whereas there was no difference in cholesterol absorption. The excretion of bile acids and lipids was also increased after consumption of oat bran compared with the control meal. In addition to the well-known hypothesis of viscous β-glucan promoting bile acid excretion (*i.e.*, entrapment of bile acids within the viscous intestinal content) and subsequent changes in the synthesis and endogenous excretion of cholesterol (*i.e.*, increased conversion of cholesterol into bile acids to compensate for the loss), the authors hypothesized a delay in the micellar lipid solubilisation process and a consequent reduction in the secretion of chylomicrons into the circulation. It is clear that processing of the oat itself, or the delivery vehicle, has the potential to significantly affect the properties and hence functionality of β-glucan. However, in many of these papers, a detailed analysis of the β-glucan component (*e.g.*, purity, content and molecular weight, and structural features such as the ratio of trisaccharide to tetrasaccharide units or the DP3/DP4 ratio) was not performed. Thus, the inconsistency between the results reported here is likely to be due to the variability in the oat matrices studied and the subsequent impact on the physicochemical properties of β-glucan and other oat components.

Finally, the simplest type of vehicle in the form of beverages (where nutrients are in contact with free water), such as milk or fruit drinks into which oat products have been incorporated, has been found, in general, to have a more regular effect on cholesterol reduction compared to more complex matrices. Consumption of these beverages systematically led to cholesterol and LDL-cholesterol reductions in all the studies reported (Table 1). However, the reductions never exceeded 9%. Considering that these are the simplest vehicles, there is less scope for physical interactions within the matrix and for improving the conditions for β-glucan hydration; therefore the physiological impact is possibly due mainly to β-glucan alone, whereas for the more complex, solid foods, interactions between β-glucan and the food matrix, for either natural or processed foods, appear to either negate or accentuate the effect. This suggests that not only is the processing of the oat β-glucan important, but the matrix or vehicle in which it is delivered is also critical for its ability to reduce serum cholesterol levels. Therefore, further research is required to understand how the physicochemical properties of the β-glucan influences its interactions with other components and structures in minimally processed (*i.e.*, where the oat tissue remains mostly intact), solid food matrices in order to fully understand the mechanisms of action and maximise the health benefits of oats.

In the majority of human studies the physicochemical or structural characteristics/properties of oat are not reported.^2^,^3^,^10^,^13^,^14^,^106^ The variabilities due to cultivars, environmental conditions, storage conditions and the processing technique applied to the oats are often ignored. The oat variety and the maturity of the grain are likely to have a significant effect on the amount and properties of the β-glucan present in the grain^32^,^107^ as well as on the starch, protein and lipid content.^1^ The quantity of β-glucan contained in the oat products given to the participants are, most of the time, estimated, and the β-glucan molecular weight not systematically measured.^2^,^3^ More importantly, the actual structure of the oat tissue and the overall food matrix used to administer the bioactive compounds (*e.g.* liquid *vs.* solid, water activity, organisation within the matrix, and degree of disruption of plant tissue) have rarely been considered.

In order to understand better the mechanisms involved, it seems essential to, firstly, have some information regarding the source and processing conditions of oats, and secondly, to fully characterise the food product(s) studied, which should include nutritional analysis but also, for example, the particle size of the oat tissue, the presence and amount of gelatinised starch, and the extent of oat cell degradation.

From the studies on the impact of oat and β-glucan-based products on plasma cholesterol in humans, it is difficult to draw firm conclusions, notably related to the potential matrix effect. Increasing the degree of matrix complexity led to conflicting results concerning its impact on lipemia (Table 1). However, the dose, study design, status (*e.g.*, hypercholesterolemic and overweight) and genetic characteristics of the subjects, food matrices, and molecular weight of β-glucans appear to greatly differ from study to study, making their comparison hazardous. Therefore, three hypotheses may be advanced but they require further investigation: (1) liquid oat-based foods seem to give more consistent, but moderate reductions in cholesterol than semi-solid or solid foods where results are more variable; (2) the quantity of β-glucan and molecular weight at the expected consumption levels (∼3 g per day) play a role in cholesterol reduction; and (3) natural β-glucan-rich oat based foods often appear to be more efficient at lowering cholesterol or lipemia than β-glucan added as an isolated ingredient.

## 3. Degree of processing and functionality

### 3.1 Effect of processing on oat grain structure and constituents

Despite being necessary for food safety, conservation, convenience, palatability and digestibility, processing can generate foods whose regular consumption has been associated with the development of chronic lifestyle related diseases (*i.e.*, obesity and cardiovascular disease)^108^–^110^ associated with a “Westernised diet”.^111^ This can be attributed to several reasons, including recent concern about the role of processing in food health potential and the very late recognition of the food matrix effect in defining its health potential,^28^ especially for cereal-based foods.^112^ These effects are usually (but not exclusively) linked to the ability of the food matrix structure to control the rate and extent of breakdown, digestion and release of nutrients.^113^ The effects can be direct, by increasing gastric volume and reducing gastric emptying, thus promoting satiation and prolonging appetite.^114^ Conversely, they can be indirect, by restricting nutrient bioaccessibility,^115^–^117^ thus delaying nutrient uptake, and hence reducing glycaemic and lipidemic responses. This can have positive benefits by promoting satiety and improving insulin response. Today we know that the populations adhering the most to highly processed foods, which are more easily digested, are the most prone to develop non-communicable diet-related chronic diseases.^118^–^124^ In reality, processing impacts both the food structure and composition,^125^ and this has to be taken into consideration for oat-based foods. The matrix effect for these foods includes physicochemical characteristics of the matrix such as nutrient interaction, matrix structure (*i.e.*, solid *vs.* semi-solid *vs.* liquid), degree of β-glucan polymerization and/or intensity of processing applied. For example, a recent study showed of 378 ready-to-eat foods that the more a food is processed, the less satiating and the more hyperglycaemic it is, with matrix effects (*i.e.*, impact of overall food structure and physico-chemical properties) playing an essential role in both outcomes.^126^,^127^ However, for oat-based foods, we are confronted with a paradoxical effect since the more products are unstructured, the more β-glucans seem available to exert a cholesterol lowering effect, but at the same time, an unstructured food (for example, when cell walls are ruptured) is likely to increase starch and glucose bioaccessibility, thus increasing glycaemia. So, it remains unresolved whether cholesterol lowering, increased satiety and decreased glycemia can be achieved in the same food.

Oat grains have to undergo some form of processing before their nutrients become digestible.^35^ Overall technological treatments may be classified into the following main groups: mechanical, thermal, enzymatic and chemical treatments as well as refining (or fractionation/recombination) and fermentation. In emerging and developing countries, malting, pre-soaking and pre-germination are also often used in traditional foods. First, the grain has to go through cleaning, grading and dehulling. The bran and germ remain on the groat after the dehulling process because the aleurone layer does not separate from the endosperm as easily in oat as it does in other cereals such as wheat.^30^ Consequently, the dehulled oat groat retains a concentrated amount of dietary fibres (*e.g.*, β-glucan) and other phytochemicals (*e.g.*, vitamins, minerals, and phenolic compounds). Naked oats, a hull free variety, have a different nutritional profile compared to dehulled oats. In general, the former contains a smaller amount of fibres but larger amounts of proteins, lipids and certain phenolic compounds than the latter.^128^,^129^ The absence of hull presents the advantage of reducing the cost for the food processing industry.^130^,^131^ During kilning, a steaming process takes place as a part of oat milling. Here, Maillard reactions occur that improve the taste of the product while promoting stability of lipid and antioxidant compounds by deactivating endogenous enzymes.^35^

Oat flakes remain the most common way to eat oat.^36^ Flakes are obtained by flattening whole or steel-cut groats. They are often partially precooked to reduce cooking time and then dehydrated.^35^ Flour can be produced by grinding the whole oat groat or flakes with a pin or hammer mill. β-Glucan is found in the cell walls, especially around the aleurone layer and outer endosperm (Fig. 1). Coarsely ground flour can then be sieved or air-classified to produce bran with an increase in the β-glucan content.

The release rate and extent, solubility and molecular weight of β-glucan when in a food product have been shown to be affected by cooking and freezing, in particular, repeated freeze–thaw cycles.^132^–^135^ It is essential to consider the storage conditions and pre-treatment (*e.g.*, hydrothermal and/or ethanol) applied to the oats, notably to prevent degradation from endogenous enzymes. The size of the oat particles and the nature of the food matrix in which they are incorporated have also been revealed to have, in *in vitro* studies, a significant impact on the rate and extent of β-glucan release.^136^,^137^ When milled oat bran was included in individual food matrices made of protein (egg white), starch (potato starch) or lipid (butter oil), protein and starch matrices led to only 5% of β-glucan release at the end of *in vitro* gastric digestion compared with ∼50% for the particles without the matrix.^136^ Hydrothermal processing generally increases β-glucan extractability;^138^ however, our recent study showed a reduced release of the polymer.^137^ This is possibly due to the cooking method used in the latter study (*i.e.*, water and oats were mixed together and then gradually brought to the boil) that promotes swelling of the cell wall and gelatinisation of the starch, hence restricting access of water to the β-glucan.^137^ Fermentation has also been found to increase the solubility of β-glucan.^139^

The methods used to extract β-glucan from oat (*e.g.*, pH, temperature, liquid to solid ratio, wet *vs.* dry milling, the particle size, enzyme treatment and the oat source) have consequences on the physicochemical properties of the polymer.^12^ The extraction process determines the yield and degree of purity of the β-glucan extract, but also its rheological behaviour since the latter is controlled by the β-glucan molecular weight, solubility, conformation and concentration.^140^,^141^ Similar to the case when it is incorporated into an oat-based product, extracted β-glucan can be depolymerised due to the storage conditions or the extraction process itself.^15^ The solubilisation of high molecular weight β-glucan can be difficult to achieve, in particular if the preparation has been freeze-dried beforehand.^142^

Germination/malting stimulates the expression of cell wall degrading enzymes,^143^ such as xylanases and glucanases, to break down the endosperm cell walls and allow access to the starch as an energy source to support germination and growth. The enzyme action will solubilise β-glucan from the cell walls and make it more available, but also the molecular weight may well be reduced.

Extrusion is a highly intensive process, simultaneously applying high pressure, shear and temperature to create food products with a range of structures and textures. Extrusion will degrade and reorganise the oat matrix into a more homogeneous structure.^144^ This may result in protein denaturation and aggregation, solubilisation and degradation of cell wall polysaccharides and the partial destruction of amylose–lipid complexes. These changes in structure and interactions are likely to affect the food digestibility and the subsequent physiological responses (*e.g.*, lipaemia, cholesterolaemia and glycaemia). However, these effects are highly dependent on processing conditions. Brahma *et al*. showed that under their conditions they could increase the amounts of resistant starch and water-extractable β-glucan by decreasing the water content during the oat extrusion, without affecting the β-glucan molecular weight.^145^ However, Tosh *et al.* also showed that drastic extrusion conditions caused β-glucan depolymerisation, loss of cell wall integrity and β-glucan dispersion throughout the cereal.^146^ In addition, alongside molecular weight reduction, differences in the hardness and density of the extruded cereals became more obvious.

Thermal treatment applied to the grain can influence the amount of soluble proteins present in oat flour and the binding sites of globular proteins responsible for binding polysaccharides.^147^,^148^ The properties of the starch granules such as their size, swelling capacity, and rheological behaviour are also altered during processing.^148^,^149^ Therefore, the processing induced changes occurring to the overall structure of the oat and the macronutrients it contains will affect the release and solubilisation of β-glucan and subsequently its effect on lipid and cholesterol metabolism.^33^,^137^,^138^,^150^

### 3.2 Effect of processing on oat functionality

As we had previously underlined, processing can influence the oat matrix (qualitative effect) and its composition (quantitative effect). As we will discuss in the next section, we are confronted with a double complexity: firstly, a single compound (*i.e.*, β-glucan) may have effects on several physiological functions, and secondly, several oat compounds may impact one defined physiological mechanism such as cholesterol reduction (see section 4).^151^ Processing, by disrupting the food matrix, facilitates the digestibility and bioavailability of nutrients but can also degrade the functionality of a food by altering the structure of its components (*e.g.*, depolymerisation of β-glucan, lipid coalescence and protein denaturation) and/or the interaction between them.

As discussed above, purified forms of β-glucan were found to be less successful at inducing changes in blood lipids^56^,^86^,^98^,^99^,^101^ than intact oat tissues.^23^,^152^ This is consistent with epidemiology studies showing that whole grain intake, but not fibre alone, is inversely associated with atherosclerotic progression and cardiovascular disease.^153^,^154^ The functionality of oats on cholesterolemia has been ascribed to the capacity of β-glucan to increase the viscosity of the intestinal content. Products containing both the bran and the endosperm of the oat (*i.e.*, oat flakes or flour) are likely to exhibit a complex rheological behaviour due to the presence of β-glucan but also proteins and starch.^137^,^149^ The rate and extent at which these nutrients are released from the food matrix and solubilised in the gastrointestinal tract will have consequences on the viscosity of the digesta and thereby their physiological activity.^15^ Very few studies measured the water activity of oats or oat products; however, water that is freely available within an oat product will dictate the solubility of the β-glucan and the interactions between oat constituents.^155^,^156^ The associations between water and other molecules is also specific to the product. The difference in water activity could be one of the reasons why differences were observed in the cholesterol lowering properties of various oat products. This is likely to be due to the competition for available water between the starch, β-glucan, and other water soluble polymers in the matrix. This will be strongly affected by processing. For example, the effect of hydrothermal processing will depend on the quantity of water present as the amount of starch gelatinisation will lead to a huge demand for water within the product, and thus affect the amount of soluble β-glucan that will be made available.

The main modification occurring to β-glucan during the processing of oat and oat-based products is depolymerisation. β-Glucan depolymerisation occurs if the β-glucanases present in the oat grains are not inactivated.^47^ Although kilning of oats is used to inactivate lipases that makes the oats rancid over time, it has the added benefit of reducing the activity of endogenous β-glucanases as well.^35^ A reduction in β-glucan molecular weight has also been observed during the production of bakery products as shown with oat breads containing wheat flour.^157^ The β-glucanases naturally present in wheat flour degrade the β-glucan during the fermentation process.^158^ The general assumption behind the cholesterol-lowering effect of oat and the β-glucan it contains is that higher β-glucan molecular weight may contribute to higher viscosity in the gastrointestinal tract and subsequently to greater plasma cholesterol reduction. Kim *et al.*^159^ suggested that the “molecular weight of β-glucan should be at least 1200 kDa in order to elicit a cholesterol-lowering impact”.^13^ Wolever *et al.* (2010) showed that extruded cereals with average molecular weights of 530 kDa were effective at doses of 3 g of β-glucan per day.^23^ However, the viscosity of the intestinal content relies not only on the molecular weight of the β-glucan, but also on its concentration, structure and behaviour in solution.^15^,^146^,^160^ Indeed, the polymer has to be present in a certain size range, at a given concentration, and dissolved in solution to form an entangled network, which then will result in an increase in viscosity. This is a complicated process that has been well described elsewhere.^161^,^162^

The inconsistency in the outcomes from human studies implies that the mechanisms are much more complex than originally thought. These findings reinforce the importance of considering the whole matrix for the delivery of bioactives. Similar effects have been found for phytochemicals other than β-glucan, where the consumption of whole fruits and vegetables seems to have a more beneficial effect than purified phytochemicals, such that the structure and delivery form are as important for functionality as the bioactive itself.^163^

## 4. Synergistic action of oat constituents

In their article published in 2009, Jacobs and co-workers clearly presented the concept of food synergy^24^ and later, in 2014, Fardet and co-workers showed the necessity for nutrition research to move towards a holistic, integrative approach.^164^ Food synergy means that the action of some nutrients and phytochemicals within a whole, natural food matrix is greater than the corresponding action of the same food constituents taken individually. In other words, synergy reflects that 2 > 1 + 1.^24^,^29^,^164^ Therefore, the quantity of a bioactive may become irrelevant if the quality of the delivering system (*i.e.*, food matrix) is ignored. A coordination exists between food components that may be lost during extensive processing; pairing/interaction is likely to be fundamental for the functionality of bioactives. More recently, the same authors discussed the arrangement of molecules within a food and showed how it is fundamental for biological functionality of the raw material.^29^ For example, β-glucans are associated with the cell walls in the outer layers of the endosperm and are thought to be involved in protection against heat or drought stress.^165^ Therefore their properties and location in the tissue during hydration and processing could be critical to their health impact during digestion. That arrangement of molecules that influences their digestibility will have physiological consequences. By applying these notions to oats and their impact on health, the following questions arise: (i) What are the compounds present in oat that exert a cholesterol lowering effect? (ii) Is the combination of these compounds enhancing their individual action? (iii) Is their organisation within the oat matrix of significance for their action?

Several compounds in oat may participate in improving lipemia, especially cholesterolemia. Thus, the potential oat lipid-lowering package includes β-glucan, arabinoxylans, polyphenols, phytic acid, phytosterols, policosanol, betaine, choline, inositols, tocopherols and resistant starch.^166^–^169^ Besides the lipid-lowering package, a group of bioactive compounds with other benefits to health may be found in oat-based products, such as antioxidants, anticarcinogenic and/or anti-inflammatory compounds.

### 4.1 Oat cholesterol-reducing agents

Oat groat is rich in a wide range of bioactive compounds, some of them present in small quantities.^43^,^167^,^169^ These bioactives may act in combination in the gastrointestinal tract, perhaps even synergistically, thus compensating for their minor contribution. Apart from β-glucan, phytosterols, phenolic compounds (*e.g.*, avenanthramides), tocols and saponins are other oat constituents that might have a direct or indirect impact on cholesterol.^43^,^168^,^170^

The beneficial impact of phytosterols on plasma cholesterol concentrations is now well documented.^171^,^172^ Phytosterols and cholesterol are structurally and functionally similar but differ in the amount they are absorbed, which is thought to contribute to reduced blood cholesterol. Some of the hypotheses regarding the mechanisms of action include displacement of cholesterol by phytosterols from the mixed micelles and formation of mixed crystals leading to the precipitation and excretion of cholesterol.^173^ Oat contains between ∼350 and 600 μg g^–1^ of phytosterols,^43^,^168^,^170^,^174^ mainly as β-sitosterol. A portion of oat (30 g) will provide on average 40 mg of phytosterols, which is much lower than the recommended ∼2 g daily intake. However, despite the low concentration found in plant foods, cholesterol reduction can be achieved following the consumption of phytosterols from natural sources, in particular when consuming whole grain products.^175^

Twenty-four phenolic compounds exhibiting antioxidant activity have been identified in oats.^36^,^168^,^176^,^177^ Their total concentration ranges between ∼200 and 870 μg g^–1^, with phenolic acids being the most common type. Phenolic compounds exist in oat either as free, soluble conjugated or insoluble bound to cell wall constituents. The form and the partitioning behaviour of these compounds influence their bioavailability and thereby their health effect.^26^ The phenolic compounds that are covalently bound to cell wall components (*i.e.*, arabinoxylan) have a low bioaccessibility compared with the free form.^178^,^179^ Avenanthramides, unique to oat, have been shown to prevent LDL oxidation; increased levels of oxidised LDL may contribute to atherosclerosis and cardiovascular disease.^180^,^181^ The majority of avenanthramides are found in oat bran (∼20–90 μg g^–1^).^43^,^168^,^177^ To a lesser extent, caffeic and ferulic acid also have antioxidant activity.^176^ When administered in combination, these phenolic acids have been shown to have a beneficial impact on the hypercholesterolemia of mice.^182^ Avenanthramides may also contribute to vascular smooth muscle cell proliferation and cell cycle progression and nitric oxide production.^183^,^184^

Tocotrienol concentrations in oat range from ∼16 to 36 μg g^–1^, primarily as α-tocotrienols (57 to 69%).^168^ In addition to their antioxidant properties, the ability of tocotrienols to reduce serum total cholesterol and LDL cholesterol levels has been demonstrated in both animals and humans.^185^ Tocotrienols seem to affect cholesterolemia by directly inhibiting cholesterol synthesis.

Saponins are amphipathic glycosides that protect plants from microbial and fungal infections.^186^ Avenacoside A and avenacoside B are two saponins only found in oats.^187^ The content of avenacoside decreases with the level of processing: oat bran and flakes being richer than ready-to-eat cereals containing processed oat products. Animal studies have revealed that saponins could interfere with cholesterol metabolism, albeit sometimes minimally.^188^–^190^ Saponins are thought to exert their cholesterol-lowering effect by forming a complex with sterols that is not absorbed by the enterocytes, thereby increasing the amount of cholesterol excretion.

For health benefits the active bioavailable dose appears to be an important parameter to consider rather than the dose ingested. It is essential to have a deep understanding of the physicochemical properties of these compounds so that their behaviour in the gastrointestinal tract during digestion can be predicted. Furthermore, their interaction(s) with other nutrients within and outside the food matrix is likely to promote their bioavailability and/or efficacy.

### 4.2 Food matrix effect and synergy

As discussed in section 3, the degree of complexity of the food matrix and its structural organisation can have an impact on health by acting on two levels, the macro- and micro-scales. On the macroscale, the structure of the food will affect how nutrients and bioactives are released, thereby influencing the rate and extent of their digestibility. Firstly, a reduced rate of nutrient digestion is likely to have health benefits related to gastric emptying, satiety, glucose and lipid metabolism.^115^,^117^ Consumption of a complex, slowly digestible food matrix would lead to a gradual release of nutrients, which will generate a moderate, controlled physiological response, for example, reduced post-prandial lipidemia and/or glycemia.^117^,^191^,^192^ Structurally complex foods may be better handled by the human body than over-processed (“pre-digested”) ones. This is because over-processed foods are often highly digestible, causing an overload of physiological cues elicited by a rapid and large release of nutrients.^127^ This nutritional overstimulation, when repeated through recurrent consumption of highly processed foods, can lead to biological malfunctions and diseases (*e.g.*, dysregulation of appetite/satiety and insulin resistance). Secondly, the reduced rate of digestion will also permit a sustained release of bioactives as observed with antioxidants.^193^,^194^ Thirdly, these bioactives could be lost during processing.^195^

These phenomena also have consequences on the microscale, where the food matrix will impact the functionality of specific compounds by modulating their bioavailability, how they are mixed and “circulated” in the gastrointestinal tract, and their absorption, or not, into the enterocytes.^196^ The food matrix could also modulate the interactions between different food compounds, which in turn could determine their delivery to the site of action (*e.g.*, lipophilic compounds requiring lipids for their transport and uptake).^197^ The molecular interactions and formation of complexes may either be essential for or detrimental to the functionality of bioactives.^193^,^194^ As a result, smaller amounts of bioactives, if they are delivered in the most effective form and/or in combination with appropriate compounds, could potentially have a greater effect than larger amounts of individual molecules.^198^

As for evidence specific to oats, animal studies support the concept of synergistic action of its components on lipemia, particularly macronutrients and β-glucan.^199^,^200^ Guo *et al.* found that five oat varieties, containing similar amounts of β-glucan and phytosterols but varying in their protein and lipid content, elicited a decrease in plasma cholesterol to different extents.^199^ The oat variety with the highest proteins and lipids content produced the greatest cholesterol reduction.

The capacity of oats to sequester bile acids has been found to be higher, presumably resulting in greater bile acid excretion for complex sources of the polymer (*i.e.*, oat flour), than purified fractions.^201^ The authors concluded that several components of the oat flour acted in synergy to elicit its hypocholesterolemic effect. Food matrices enriched with both β-glucan and sterols from mushrooms (ergosterols) also showed an enhanced displacement of cholesterols from micelles compared with ergosterols on their own.^202^ Another study performed on pigs revealed that the consumption of oat β-glucan influenced the absorption of neutral sterols at different sites of the small intestine and in part of the colon.^19^

Because phytosterols have limited solubility both in water and lipid, their bioavailability and efficacy rely on many factors, such as the quantity of lipid, the type of phytosterol (*i.e.*, sterol, stanol or ester such as steryl glycoside), the source and the food microstructure.^203^,^204^ Similarly to β-glucan, the effect of phytosterols on blood cholesterol and cholesterol metabolism depends on the food matrix (“vehicle” that delivers the bioactives).^205^,^206^ Natural matrices, such as minimally processed oat, may be an ideal form for the delivery of phytosterols. The mixture of free (*i.e.*, sterol or stanol) and bound (esters) phytosterols found in plant food,^207^ associated with various nutrients, may facilitate their transport in the gastrointestinal tract and their delivery to their site of action.

Polyphenols when associated with dietary fibres have been shown to reduce cholesterol levels.^208^ The material that survives gastrointestinal digestion and gets delivered to the colon may promote the growth of bacteria having a positive effect on blood cholesterol concentrations, notably *via* bile salt hydrolase activity.^209^–^211^

It seems reasonable to venture the hypothesis that the proportion of compounds present in oat, and the complex associations between them, influence the bioavailability and activity of the bioactives. A thorough analysis of the literature on the cholesterol-lowering effect of oat, together with current knowledge of the food structure, suggests that the concerted action of the oat constituents, delivered in an optimal amount in relation to each other, could enhance the functionality of oat and elicit a positive, or balanced, physiological response. Future work in this area should not focus solely on individual/isolated nutrients that are removed from their “food context”, but also incorporate detailed, well characterised studies of the food matrix that contains the nutrients in order to shed some light on the mechanisms behind the positive health effects of oat. Both *in vitro* and *in vivo* studies clearly demonstrated the beneficial effect of oat on cholesterolemia, which is unlikely to be due exclusively to β-glucan, but rather to a combined and synergetic action of several oat compounds acting together to reduce blood cholesterol levels.

## 5. Conclusion

One of the main challenges faced today by the industry is to find the right balance between none or little food processing and high levels of food transformation. As discussed above, oat requires to be processed to make it safe to consume but not to such an extent that it loses its nutritional values and processing become detrimental. A distinction ought to be made between oat-based foods according to their degree of processing,^123^ which will help identify the foods with optimal health benefits.

Using whole or complex fractions of oat rather than isolated/purified phytochemicals could be profitable for the industry given that less processing and extracting agents/solutions would be necessary. In order to avoid fluctuations between batches and to ensure a high quality of the products, detailed characterisation is fundamental to define the ranges of bioactivity. These foods also have to be attractive to consumers if the health impacts are to be fully realised, which is another challenge to the food industry. Further research should therefore aim at identifying the most active forms of oat and the combination of nutrients and phytochemicals in relation to cholesterol levels and risk of cardiovascular disease. A better characterisation of the structural form of oat that is ingested and as it progresses through the gastrointestinal tract is still required to provide valuable information for the design of healthier food products.

## Conflicts of interest

The authors are not aware of any affiliations, memberships, funding, or financial holdings that might be perceived as affecting the objectivity of this review.
